# Transient Replication of Hepatitis C Virus Sub-Genomic RNA in Murine Cell Lines Is Enabled by miR-122 and Varies with Cell Passage

**DOI:** 10.1371/journal.pone.0089971

**Published:** 2014-02-26

**Authors:** Patricia A. Thibault, Joyce A. Wilson

**Affiliations:** Department of Microbiology and Immunology, and Vaccine and Infectious Disease Organization-International Vaccine Centre, University of Saskatchewan, Saskatoon, Saskatchewan, Canada; University of Washington, United States of America

## Abstract

Hepatitis C Virus (HCV) is a serious global health problem, infecting almost 3% of the world’s population. The lack of model systems for studying this virus limit research options in vaccine and therapeutic development, as well as for studying the pathogenesis of chronic HCV infection. Herein we make use of the liver-specific microRNA miR-122 to render mouse cell lines permissive to HCV replication in an attempt to develop additional model systems for the identification of new features of the virus and its life cycle. We have determined that some wild-type and knockout mouse cell lines – NCoA6 and PKR knockout embryonic fibroblasts – can be rendered permissive to transient HCV sub-genomic RNA replication upon addition of miR-122, but we did not observe replication of full-length HCV RNA in these cells. However, other wild-type and knockout cell lines cannot be rendered permissive to HCV replication by addition of miR-122, and in fact, different NCoA6 and PKR knockout cell line passages and isolates from the same mice demonstrated varying permissiveness phenotypes and eventually complete loss of permissiveness. When we tested knockdown of NCoA6 and PKR in Huh7.5 cells, we saw no substantial impact in sub-genomic HCV replication, which we would expect if these genes were inhibitory to the virus’ life cycle. This leads us to conclude that along with the influence of specific gene knockouts there are additional factors within the cell lines that affect their permissiveness for HCV replication; we suggest that these may be epigenetically regulated, or modulated by cell line immortalization and transformation.

## Introduction

Hepatitis C is a blood-borne viral disease prevalent worldwide; current estimates suggest approximately 150 million people are infected [1]. Hepatitis C virus (HCV) becomes chronic in approximately 70% of acute infections, and can lead to the development of various cancers, liver steatosis, and numerous other complications throughout infection [2]. A member of the Flaviviridae family, HCV is an enveloped virus with a single-stranded RNA genome of positive orientation, and is a member of the genus *Hepacivirus*. The HCV genome is approximately 9.6 kb in length, and consists of an uncapped 5′ un-translated region (UTR) containing significant secondary RNA structure deemed essential for viral RNA replication and an internal ribosomal entry site (IRES) essential for translation, followed by a single open reading frame (ORF) containing all the viral genes, and completed by a 3′ UTR with further secondary RNA structures necessary for genome replication [3]. The single ORF of the virus is translated as a polyprotein that is then cleaved into the individual viral proteins by both cellular and viral proteases [3].

At least two aspects of the viral life cycle are major determinants of host range and cell specificity for Hepatitis C Virus. Entry factors appear to define host range of the virus, as has been demonstrated both *in vitro* and *in vivo*: addition of human CD-81 and human Occludin to murine cells permits entry of the virus, while generation of mice transgenic for these factors has permitted infection [4], [5]. However, these and other identified receptors – SR-B1, Claudin-1, TfR1, NPC1-L1, Syndecan-1, EGFR, and EphA2– do not appear to define the liver specificity of HCV, as they can be found on and in many other human cell types as well [4]–[14]. ApoE expression (murine or human), which is liver-specific, does appear to be a requirement for production of infectious virions following infection, but is not required in the host cell for earlier stages of entry or RNA replication [4], [15], [16].

A host factor that likely influences liver-specificity of HCV replication is miR-122, a liver-specific microRNA that binds to two sites on the 5′ UTR of the HCV genome [17]–[19]. This miRNA is highly abundant in the liver, accounting for approximately 70% of the small regulatory RNAs in hepatocytes; while its role in the liver is not yet completely defined, it does appear to regulate typical aspects of liver cell function such as cholesterol production and secretion, and it is also implicated as a tumour-suppressor microRNA [20]–[23]. Interestingly, we and others have shown that expression of miR-122 in human liver cell lines previously considered refractory to HCV replication renders the cells permissive to HCV replication [24]–[26]. This has also been demonstrated in other non-human and non-liver cell lines, mostly through use of stable replicons rather than transient replication [27]–[29].

There is a need for more and better model systems in HCV research. Although we have recently begun adding other human and hepatocyte-derived cell culture systems to the predominant Huh7-derived cell lines, non-human – particularly murine – cell lines remain a critical stepping-stone to animal model development. A series of efforts using knockout mouse cells, selectable virus replicons, and human factor transduction has recently culminated in multiple research groups achieving the complete virus life cycle in mouse cells expressing the required entry and liver factors [4], [15], [30], but animal models of HCV infection are still limited [31]. HCV mouse models relying on SCID-uPA mice supporting xenografted human liver tissue can be infected, and virus-induced pathogenesis or drug response studied, but the mice have limited life spans, lack immune systems, and are expensive to produce [32]. Similar mice have only recently been developed to contain a humanized immune system, but the expense of these animals, as well as the fact that their livers and immune systems come from humans and vary between each xenografted mouse, remains an obstacle for their widespread use in HCV studies [33], [34]. Most recently, the human CD-81/Occludin transgenic mouse model developed by Dorner *et al.* has been demonstrated to support the entire viral life cycle, but since it relies on innate immune gene knockouts such as Stat1 or IFNα/β, it is as yet unknown how much of the immune-mediated pathology will be recapitulated [6]. The chimpanzee best represents the disease in humans, but they are currently not available for most research, and present both ethical and financial obstacles [31], [35], [36]. Tree shrews (*Tupaia belangeri*) have also been identified as a potential model for HCV chronicity and pathogenesis, but they have also not yet been evaluated for immunological aspects of the infection [37]–[39].

Thus, we chose to examine mouse cell lines to identify other genetic knockouts that could render mouse cells, and potentially the mice of origin, permissive to HCV replication. We identified one wild-type and two knockout mouse embryonic fibroblast (MEF) cell lines, “GH” wild-type, and NCoA6 and PKR knockout cells, that were permissive to transient, unselected sub-genomic HCV RNA replication when supplemented with miR-122. GH wild-type MEFs permitted only low levels of sub-genomic HCV RNA replication, while PKR MEFs initially supported high levels of sub-genomic RNA replication, but later passages, alternate isolates, and alternate sources of PKR knockout cells did not display a permissive phenotype. NCoA6 knockout cells also supported sub-genomic HCV RNA replication, although not to the levels seen in PKR MEFs, and HCV RNA replication in these cells also varied with cell passage. Since PKR and NCoA6 knockdown in Huh7.5 cells had only a minor impact on sub-genomic RNA replication, we conclude that the permissiveness of our mouse cell lines, while possibly modulated by the gene knockout, was also defined by other aspects of the cell. We speculate that cell line modifications induced by culturing, immortalization, transformation, or other epigenetic changes also impact permissiveness of cells to HCV replication.

## Results

### One Wild-type MEF Cell Line is Permissive to HCV Replication When Supplemented with miR-122, While Others are Not

**Figure 1:**
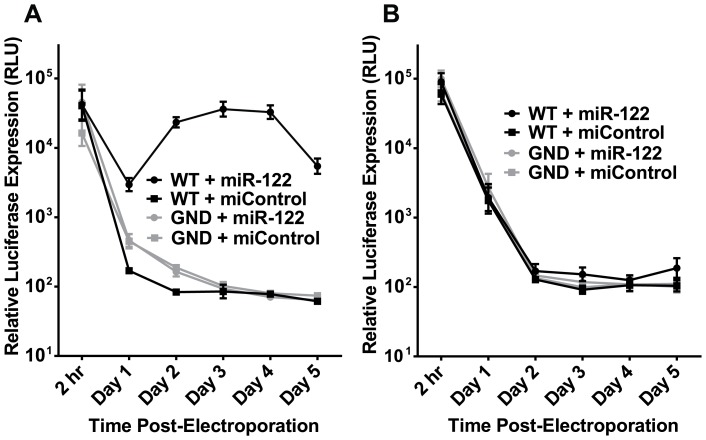
Mouse cells with no known knockouts have varying permissiveness to HCV replication when supplemented with miR-122. **A)** Wild-type MEFs obtained from Gregory Hannon were electroporated with either wild-type (WT) or replication-incompetent (GND) sub-genomic HCV RNA and the indicated miRNA (miControl, or miR-122), and luciferase expression was monitored at the indicated time points as a measure of HCV replication. **B)** Wild-type MEFs obtained from Per Antonson were treated as indicated in (A). Results from (A) and (B) are displayed as mean and standard error of the mean (SEM) of three or more independent experiments.

Wild-type MEFs from multiple sources were tested for permissiveness to HCV replication by electroporating cells with either wild-type (WT) or replication-incompetent (GND) sub-genomic HCV RNA, and supplementing the cells with either a control miRNA (miControl) or miR-122. Since the HCV RNA expresses a firefly luciferase reporter gene, replication was monitored by assaying firefly luciferase expression; firefly reporter luciferase expression from the wild-type viral RNA was compared to the non-replicating polymerase GND mutant to confirm replication, and electroporation efficiency was similar in all cases as determined by co-electroporation of a Renilla luciferase mRNA (data not shown). The MEFs depicted in Figure 1A were permissive to low levels of sub-genomic HCV RNA accumulation when electroporated with synthetic miR-122; however, the raw luciferase expression was approximately a thousand-fold lower than ia typical in Huh7.5 cells, which was reflected in our inability to detect RNA accumulation via northern blot (data not shown). In a different wild-type MEF cell line, sub-genomic HCV RNA was unable to replicate in any detectable manner regardless of miR-122 supplementation (Figure 1B). In fact, of the five wild-type MEF cell lines we tested, as well as commonly-available NIH 3T3 cells (data not shown), only those shown in Figure 1A demonstrated detectable HCV replication. We therefore conclude that some wild-type MEFs are permissive to low levels of transient sub-genomic HCV replication when supplemented with miR-122. As we later observed with other knockout mouse cell lines, permissiveness of these MEFs varied through passage, although not in a predictable manner.

### PKR Knockout MEFs are Permissive to High Levels of Sub-genomic HCV RNA Replication when Supplemented with miR-122, but did not Maintain Their Phenotype Between Passages and Isolates

**Figure 2:**
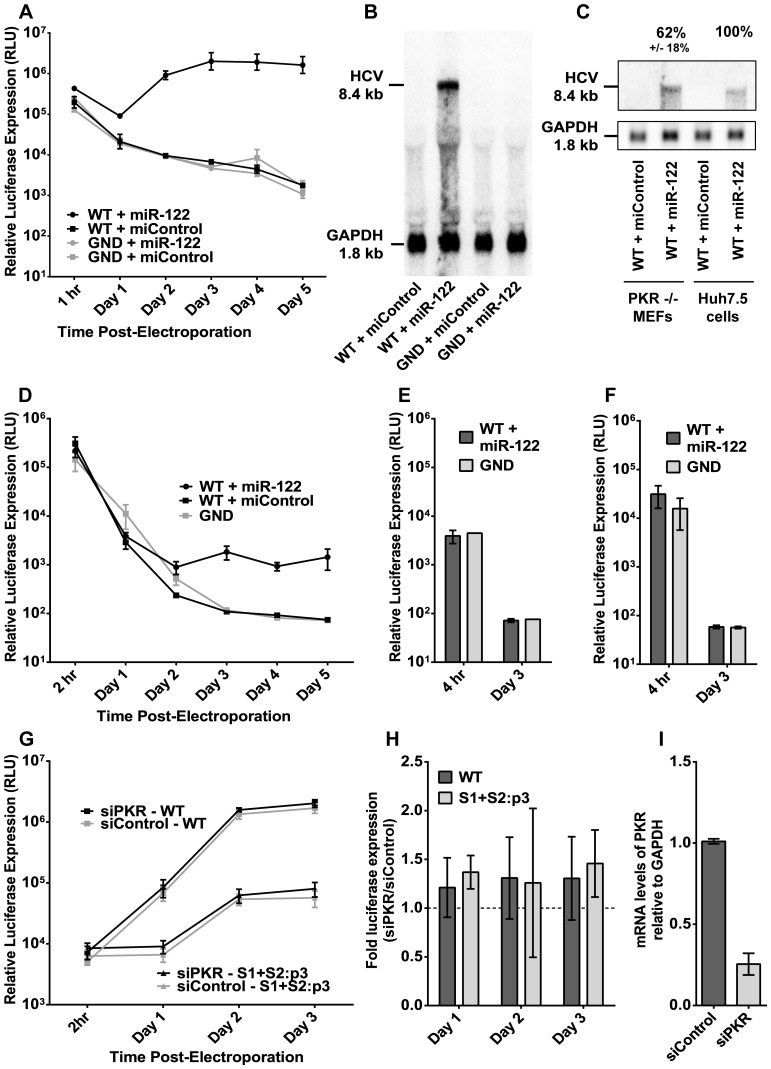
PKR knockout MEFs are permissive to sub-genomic HCV RNA replication when supplemented with miR-122. **A)** PKR knockout MEFs were treated as in Figure 1, and sub-genomic HCV replication was monitored at the indicated time points via luciferase expression. Results are shown with SEM, and are an average of two independent experiments. **B)** RNA from (A) was isolated from PKR knockout MEFs three days post-electroporation and probed for HCV RNA accumulation by northern blot, with GAPDH as a loading control. **C)** RNA from (A) was compared with RNA from Huh7.5 cells via northern blot. Bands from two independent experiments were quantified by densitometry, and normalized to GAPDH. Percentage HCV RNA +/− standard deviation is presented relative to Huh7.5 cells. **D)** Primary MEFs isolated from PKR knockout mice were treated as in Figure 1. Results are shown with SEM and are an average of nine independent experiments. **E)** Primary hepatocytes were isolated from PKR knockout mice and electroporated with sub-genomic RNA and miR-122. Luciferase expression was evaluated four hours post-electroporation to assess RNA transfection efficiency and three days post-electroporation to detect sub-genomic RNA replication. Reporter activity above non-replicating (GND) levels was not detected. Results are an average of four experiments, shown with standard deviation. **F)** Primary hepatocytes isolated from PKR knockout mice were transfected via Lipofectamine 2000 with sub-genomic RNA and miR-122, and evaluated as in (E). Reporter activity above non-replicating (GND) levels was not detected. Results are an average of six experiments, shown with standard deviation. **G)** Huh7.5 cells were first electroporated with the indicated siRNAs (siControl or siPKR), three days before a second electroporation (Day 0) with the indicated sub-genomic HCV RNA (wild-type (WT) or S1+S2:p3) and a second dose of siRNA. Results are an average of six independent experiments, shown with SEM. Experiments were analyzed with a two-way repeated measures ANOVA, and differences with siRNA treatment were not found to be statistically significant. **H)** The effect of PKR knockdown on HCV replication in Huh7.5 cells was evaluated by examining the fold-change in replication over siControl-treated cells that is caused by siPKR treatment. Error is shown as the standard deviation. **I)** PKR knockdown in Huh7.5 cells was confirmed by qRT-PCR, and is shown as an average of three experiments with standard deviation.

Protein kinase R (PKR) is a known anti-viral protein in mammalian cells [40], and PKR knockout MEFs were reported to support colony formation with selectable sub-genomic HCV more efficiently than wild-type MEFs [4], [41]. Thus we hypothesized that PKR knockout MEFs would support efficient transient HCV replication if supplemented with miR-122. Upon electroporation of primary PKR knockout MEFs acquired from John Bell [42] with sub-genomic HCV RNA and miR-122 (Figure 2A), we observed high levels of luciferase reporter expression, comparable to the levels achieved in Huh7.5 cells, the most common cell line used for HCV research. Northern blot analysis confirmed sub-genomic HCV RNA accumulation in these PKR knockout MEFs (Figure 2B), and levels were within 2-fold of those observed in Huh7.5 cells, which verified that luciferase expression accurately reflected HCV RNA accumulation (Figure 2C).

These data were generated from two replicate experiments with a single isolate of primary PKR MEFs that we now believe to have been different from other PKR MEFs we tested. When further replicates were attempted with other isolates of PKR MEFs, we were unsuccessful in achieving detectable replication (data not shown), including PKR knockout MEFs obtained from Robert Silverman, which had been shown previously to support HCV replicon colony formation [43]. Moreover, primary MEFs (Figure 2D) that we isolated from PKR knockout mice could support only low levels of HCV replication in the presence of miR-122, and primary PKR knockout hepatocytes did not support detectable HCV replication at all (Figure 2E and 2F), despite efficient RNA transfection (based on luciferase expression 2 and 4 hours post-transfection) by both electroporation (Figure 2E) and transfection (Figure 2F). Since PKR MEFs are visually distinct from Huh 7.5 cells, the only other cell line in use in our lab at the time, and our PKR MEFs did not replicate HCV in the absence of miR-122 (Figure 2A, WT+miControl), we omit contamination of the MEF cell line with Huh 7.5 cells as a possible explanation for positive results shown in Figure 2A. Therefore we show that a single isolate of PKR knockout MEFs was highly permissive for transient HCV RNA replication when supplemented with miR-122.

To determine the influence of PKR on cell permissiveness for HCV, we tested the effects of PKR knockdown in Huh7.5 cells. Huh7.5 cells were electroporated with siRNA to PKR (siPKR) or to a control sequence (siControl) and allowed three days for the knockdown to take effect. The cells were then electroporated again with the indicated siRNA, as well as wild-type (WT) sub-genomic HCV RNA or miR-122 binding site mutant (S1+S2:p3) sub-genomic RNA (Figure 2G). Because Huh7.5 cells already express miR-122, it was not added in this assay. The S1+S2:p3 RNA was tested because it is not responsive to miR-122, but is capable of replicating at low levels without miRNA supplementation, and so may be more sensitive to removal of anti-viral factors because the cell’s machinery is not saturated. Both wild-type and S1+S2:p3 sub-genomic luciferase reporter expression was increased between 1.2 and 1.5-fold by knockdown of PKR (Figure 2H), but this increase was not significant when tested by two-way ANOVA (Figure 2G). Treatment of Huh7.5 cells with siPKR reduced PKR mRNA levels by 75% as measured by qRT-PCR (Figure 2I). Because knockdown of PKR in Huh7.5 cells has no significant impact on HCV RNA replication, we suggest that the permissive phenotype of the particular isolate of PKR knockout MEFs depicted in Figures 2A, B, and C may not be due to the genetic knockout, and may instead be due to other factors unique to that isolate.

### NCoA6 Knockout MEFs are Permissive to Sub-genomic HCV RNA Replication Upon Supplementation with miR-122, but do not Maintain Their Phenotype Between Passages

**Figure 3:**
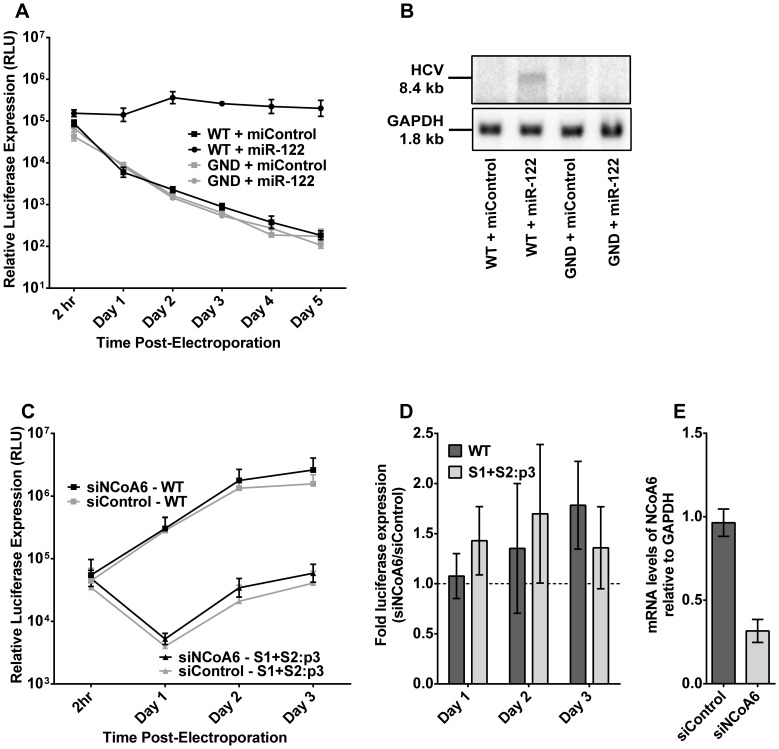
NCoA6 knockout MEFs are permissive to sub-genomic HCV RNA replication upon supplementation with miR-122. **A)** NCoA6 knockout MEFs were treated as in Figure 1, and sub-genomic HCV replication was monitored at the indicated time points via luciferase expression. Results are an average of four experiments, shown with SEM. **B)** RNA from (A) was isolated from NCoA6 knockout MEFs three days post-electroporation and evaluated for HCV RNA accumulation by northern blot, with GAPDH as a loading control. **C)** Huh7.5 cells were first electroporated with the indicated siRNAs (siControl or siNCoA6), three days before a second electroporation (Day 0) with the indicated sub-genomic HCV RNA (Wild-type (WT) or S1+S2:p3) and a second dose of siRNA. Replication was evaluated by luciferase expression. Results are an average of five independent experiments, shown with SEM. Experiments were analyzed with a two-way repeated measures ANOVA, and differences with siRNA treatment were not found to be statistically significant. **D)** The effect of NCoA6 knockdown on HCV replication in Huh7.5 cells was evaluated by examining the fold-change in replication over siControl-treated cells that is caused by siNCoA6 treatment, and are shown with standard deviation. **E)** NCoA6 knockdown in Huh7.5 cells was confirmed by qRT-PCR in an average of three independent experiments, and is shown with standard deviation.

Nuclear co-activator 6 (NCoA6, also known as RAP250, TRBP, NRC, ASC2, and PTIP) is a member of the nuclear receptor co-activator family, and aids in activation of the liver X receptor (LXRα) [44]–[46]. This leads to transcription of genes involved in cholesterol metabolism and export, which may impact HCV replication and production of virus particles [46]. NCoA6 was also detected in a large-scale siRNA screen to negatively impact HCV replication, but is not associated with major innate immune deficiencies [47]. We therefore acquired NCoA6 knockout MEFs from Per Antonson to test this knockout for permissiveness to HCV replication [48]. When electroporated with sub-genomic HCV and miR-122, these cells demonstrated levels of luciferase (Figure 3A) and HCV RNA (Figure 3B) that, while robust, were not as high as those detected in PKR knockout MEFs, nor in Huh7.5 cells (data not shown). The knockout is embryonic-lethal, so we were unable to acquire mice and generate hepatocytes to test for additional permissiveness [48]. In addition, the NCoA6 knockout MEFs displayed varying levels of permissiveness to HCV RNA replication, with no clear correlation between isolate, passage number, or experiment date; data shown here is from strongly-permissive cells, and we did eventually exhaust our stocks of permissive cells.

As we did for PKR, we also tested the NCoA6 knockdown phenotype in Huh7.5 cells via siRNA knockdown. Knockdown of NCoA6 increased luciferase expression from both miR-122-dependent (WT) and miR-122-independent (S1+S2:p3) sub-genomic replicons (Figure 3C) 1.4 to 1.8-fold at days 2 and 3 post-second electroporation (Figure 3D), but the effect of siNCoA6 treatment was not statistically significant as determined by a two-way ANOVA. siNCoA6 treatment of Huh7.5 cells resulted in a 68% reduction in NCoA6 mRNA levels in comparison to siControl treatment, relative to GAPDH mRNA (Figure 3E). As NCoA6 knockdown also has no significant impact on HCV RNA replication in Huh7.5 cells, we suggest that, as we found with the PKR knockout cells, the knockout of NCoA6 in the MEFs may not be responsible for the permissive phenotype of these cells.

### NCoA6 Knockout MEFs are not Permissive for Full-length HCV RNA Replication

**Figure 4:**
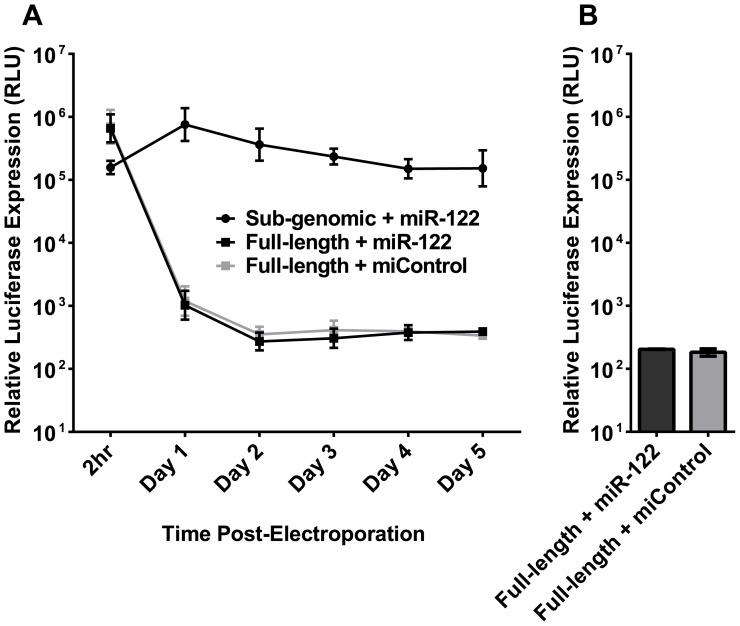
NCoA6 knockout MEFs are not permissive for full-length HCV RNA replication. **A)** NCoA6 knockout MEFs were electroporated with full-length HCV RNA or sub-genomic HCV RNA and the indicated miRNA. Note that sub-genomic HCV RNA uses a firefly luciferase reporter, while full-length HCV RNA uses a Renilla luciferase reporter, and the relative light units measured (RLU) are not directly comparable. **B)** Supernatant from NCoA6 knockout MEFs electroporated with full-length HCV RNA was harvested five days post-electroporation and plated on Huh7.5 cells, which are permissive for HCV infection and replication without additional modification. Three days post-infection, the Huh7.5 cells were monitored for luciferase expression as an indication of replication. The luciferase levels indicated here are equivalent to background. Results in (A) and (B) are shown with SEM and are an average of three independent experiments.

To further investigate the possibility of using knockout MEFs as a model for HCV replication, we examined NCoA6 knockout cells’ permissiveness for full-length HCV RNA replication. The cells were electroporated with J6/JFH-1(p7-Rluc2a), a full-length mono-cistronic HCV RNA construct that bears the virus’ structural proteins and a Renilla luciferase reporter. Both wild-type (FL WT) and replication-deficient (FL GNN) full-length HCV RNAs were electroporated with either miControl or miR-122 (Figure 4A). Although sub-genomic HCV RNA, included as a positive control, showed the cells to be permissive for replication of HCV RNA, and a firefly luciferase mRNA was included to confirm consistent electroporation efficiency (data not shown), full-length HCV RNA did not replicate detectably when supplemented with miR-122. In addition, when supernatant was collected from the MEFs on Day 5 and transferred to susceptible Huh7.5 cells, no infectious virus was detected (Figure 4B). In our hands, luciferase expression in reporter virus infected cells is sensitive enough to detect a single HCV focus-forming unit (FFU; data not shown), indicating that the full-length RNA did not produce infectious virus in NCoA6 knockout MEFs. Therefore, NCoA6 cells, while competent for sub-genomic replication, do not permit replication of full-length HCV RNA or production of infectious virus particles.

### Sub-genomic HCV RNA Replication is Unaffected by Co-electroporated Full-length RNA

**Figure 5:**
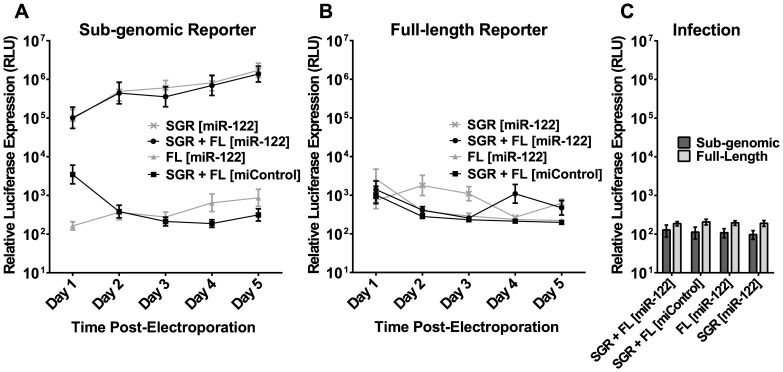
The inability of full-length HCV RNA to replicate in NCoA6 knockout MEFs cannot be complemented by sub-genomic HCV RNA, and sub-genomic RNA is not packaged into particles when co-electroporated with full-length HCV RNA. NCoA6 knockout MEFs were electroporated with sub-genomic and/or full-length HCV RNA as indicated, and either miControl or miR-122. **A)** Sub-genomic HCV RNA encodes a firefly luciferase reporter that was monitored simultaneously with **B)** the full-length HCV RNA Renilla luciferase reporter in these cells at the indicated time points. **C)** Supernatant from the cells in (A, B) was collected three days post-electroporation and plated on permissive Huh7.5 cells to test for infectivity. Three days post-infection, Huh7.5 cells were monitored for both firefly (dark grey, Full-length) and Renilla luciferase (light grey, Sub-genomic) reporter activity to detect either production of infectious particles containing trans-packaged sub-genomic HCV RNA or full-length HCV RNA respectively. Luciferase levels indicated here are equivalent to background. Results in (A), (B), and (C) are shown with SEM and are an average of five independent experiments.

One explanation for the inability of full-length HCV RNA to replicate in NCoA6 MEFs is that the full length RNA, or structural proteins expressed by it, activate murine innate immune sensors in the mouse cells, leading to induction of an anti-viral response. To test this we co-electroporated NCoA6 knockout MEFs with sub-genomic and full-length HCV RNA simultaneously, with the expectation that if the full length HCV genomic RNA was inducing an antiviral response in the host cell, then replication of the co-electroporated SGR RNA will be negatively influenced by the presence of full length HCV RNA. Because electroporation results in a >95% efficiency of RNA transfection in MEFs (data not shown), we were confident that the majority of cells would receive both viral RNAs, as well as the indicated miRNA. The sub-genomic construct encodes firefly luciferase (Figure 5A), while the full-length construct encodes Renilla luciferase (Figure 5B); each luciferase utilizes a different substrate, and so their expression can be evaluated separately within the same sample. There was no indication of *trans*-inhibition of SGR replication by full-length HCV RNA, since replication of sub-genomic RNA was not impacted by addition of full-length RNA (SGR+FL [miR-122], Figure 5A), and replication levels were similar to those seen in cells electroporated with SGR RNA alone (SGR [miR-122], Figure 5A). As expected, no sub-genomic RNA reporter activity was identified in the sample that contained only full-length RNA (FL [miR-122]), and no sub-genomic replication was detected in cells given sub-genomic RNA without miR-122 (SGR [miControl]). This led us to conclude that the full-length RNA did not activate a *trans*-acting anti-viral response, nor cause any changes in the cell that could affect sub-genomic RNA replication. Due to the design of the assay, when firefly (sub-genomic) luciferase levels are very high, some signal will bleed through into the Renilla (full-length) measurement even when no full-length RNA is present (SGR [miR-122], Figure 5B). No detectable full-length replication was observed in any of the samples (Figure 5B), showing that sub-genomic RNA was also unable to *trans*-complement full-length replication. In addition, tissue culture supernatant from these experiments demonstrated no detectable infectivity in Huh7.5 cells (Figure 5C), showing that the full-length RNA also did not produce sufficient levels of structural protein to *trans*-package the sub-genomic HCV RNA [49].

## Discussion

Here we demonstrate that supplementing non-permissive mouse cells with miR-122 can render them permissive to transient sub-genomic HCV RNA replication; we bypass the human entry factor requirements by electroporating cells with viral RNA, rather than infecting them. miR-122 is a liver-specific co-factor that is important for HCV RNA replication, and has thus been implicated in defining tissue tropism for the virus, and we confirm this as its addition renders murine embryonic fibroblasts permissive to HCV RNA replication. We identified wild-type and knockout (PKR and NCoA6) mouse embryonic fibroblasts that were permissive to detectable levels of HCV RNA replication when supplemented with miR-122. In general the gene knockouts increased the levels of HCV replication above that seen in wild-type MEFs and suggest that part of the permissive phenotype is mediated by the gene knockout as has been shown by others [4], [6], [15], [29], [30]; however, we note that alternate isolates of wild-type and knockout MEFs were not permissive to HCV replication, and the permissiveness of each cell line further varied by passage and isolate. This variation was particularly observed with PKR knockout MEFs and hepatocytes from different isolates and multiple backgrounds (Figure 2), but was also observed with the wild-type and NCoA6 knockout MEFs through cell passage. In addition, when evaluating knockdown of either PKR or NCoA6 expression in the already-permissive Huh7.5 cells in the context of sub-genomic replication, we observed an insignificant pro-viral effect that we deemed unable to account for the permissiveness of the knockout in mouse cells, which leads us to suggest that at least part of the permissive phenotype of the knockout cell line is not directly due to the particular gene that is abolished. This, however, does not preclude the possibility that there are host and cell-type specific factors that may explain the lack of effect of knockdown in Huh7.5 cells. HCV, while adept at circumventing human host defense proteins such as PKR, could still be susceptible to murine PKR and this may be one of the reasons that the PKR knockout MEFs did originally permit high levels of replication. It is also possible that the absence of NCoA6 in non-hepatic cells (fibroblasts) had a more drastic effect than knockdown of NCoA6 in the liver-derived Huh7.5 cells, as modulation of lipids in liver-derived cells may be more nuanced or have more redundancies active than in fibroblasts; it is also possible that there is a species-specific effect of NCoA6 as we speculate with PKR. Ultimately, the loss of permissiveness indicates that there are factors other than the gene knockouts involved in permitting HCV replication in these cells.

Overall, we conclude that the permissiveness of a given MEF line may be influenced by both the targeted gene knockout, and genetic and/or epigenetic changes in the cell line that occur during transformation and/or immortalization. This is based on the varying permissiveness of different cell isolates and passages of the same knockout cell line, and our finding that the HCV permissive phenotype of an isolate of primary PKR knockout cells was not observed in other primary MEF isolates and hepatocytes. We concede that there may be some basic genetic differences in anti-viral responses dependent upon the background of the mouse from which the MEFs were isolated, but the variability in permissiveness of immortalized MEFs over different passages suggests that this is not a key factor. Lohmann *et al.* noted that Huh7 cells themselves vary in permissiveness to HCV by up to 100-fold at different passages, so it is perhaps unsurprising to also observe this in other immortalized cell lines [50]. Thus, we suggest that future research with HCV in immortalized knockout cell lines be undertaken with caution and effort to confirm the effect of the knockout on HCV replication in primary cells, and to develop a means of characterizing and retaining the susceptible phenotype of the cultured cells.

Recently, Vogt *et al*. used drug selection to maintain sub-genomic and full-length HCV RNA replication in MEFs [4]. They also concluded that the use of antiviral gene knockouts increased the ability to select for stable cells. Based on these results we speculate that in both our study and in theirs that a subset of cells in each MEF population support HCV replication, and that their use of drug selection reinforced maintenance of the susceptible subset while eliminating cells that lost permissiveness through passage. Thus it is possible that in our studies, cells that support HCV replication were lost from the cell population. Unlike our study however, in the Vogt report and other previous reports, miR-122 was not required for stable HCV colony selection [4], [28], [29], [41], [51], [52]. This suggests that perhaps the knockout cells supported sufficient replication independent from miR-122 for selection (or generation of viral mutants that do not require miR-122), or that the selected cells could express miR-122 or other pro-viral factors. More recently, the same group has demonstrated the complete virus life cycle in entry-factor transgenic mice, although these mice also benefit from the same immune gene knockouts identified in stably selected MEFs [4], [6]. Two other recent publications have shown replication of HCV in MEFs, primary murine hepatocytes, and murine hepatoma cells from innate immune knockout mice in the absence of selection [15], [30]; the wild-type and NCoA6 knockout cell lines we used here were immortalized through crisis, during which any number of changes may have occurred, and therefore we cannot directly compare them to the passage status and immortalization of the cell lines established by Frentzen *et al.* and Nandakumar *et al*.

Our findings that NCoA6 knockout MEFs do not support detectable transient replication of full-length HCV RNA also require consideration. We originally considered these cells as a control knockout cell line until we determined that replication was higher in these cells than in the wild-type MEFs; we then hypothesized that a MEFs cell line could be made to support HCV replication through knockout of a host gene that does not affect innate immunity. However, the finding that NCoA6 knockout MEFs were not permissive for full-length HCV RNA replication limits their usefulness as models. Why the cells could not replicate full length HCV genomic RNA remains to be determined, but elements of the structural gene region may be involved since Aly *et al.* noted a difference in permissiveness of mouse cell lines for HCV replication that varied by the structural gene region of the construct used [53]. In our hands, the full-length RNA did not appear to affect co-electroporated sub-genomic replication, arguing that whatever prevents full-length replication in the cells does so in *cis* and is not, therefore, a dominant antiviral response triggered only by full-length HCV RNA. This is supported by others’ data that mouse cells do not restrict replication of HCV when fused with permissive human liver cells [54]. Thus, neither expression of the structural proteins, nor structural protein gene regions of the RNA, promotes an antiviral state in the cells, since neither protein nor RNA adversely affected replication of sub-genomic HCV RNA in the same cell. This suggests another, non-immune limiting factor present in this particular cell line – and perhaps others – that could be exploited in place of innate immune knockouts.

There are two major differences between the sub-genomic and full-length HCV RNAs that could affect replication in *cis*: the first is the structural protein coding region in the full-length RNA, while the second is the presence of the second (EMCV) IRES in the sub-genomic RNA construct. It is possible that the structural protein coding region contains *cis-*acting regulatory sequences that regulate HCV replication, and that a human specific host factor, absent from mouse cells, is required to activate this regulatory element. On the other hand, in the sub-genomic construct, the EMCV IRES drives expression of the non-structural proteins required for RNA replication instead of the HCV IRES. If the EMCV IRES drives translation more strongly than the HCV IRES in mouse cells, then it may lead to more efficient expression of the viral non-structural proteins required for replication and this may overcome a host species barrier in the presence of miR-122. Finally, it may be a combination of these two factors, or the presence of the EMCV IRES may disrupt long-range *cis* regulatory elements in the non-structural genes in any host cell, permitting de-regulated replication of the sub-genomic RNA in any permissive environment.

We therefore suggest that there are additional factors that need consideration when attempting to develop non-human cell culture models for HCV; the immortalization and subsequent maintenance regime of the cells may affect their permissiveness in addition to any knockout background, particularly when attempting unselected HCV RNA replication. Furthermore, the viral construct may also be important when attempting to identify permissive cell lines as was seen in the work by Aly *et al.* [53]. Future work with new viral isolates may also advance the search for new murine models of Hepatitis C Virus.

## Materials and Methods

### Ethics Statement

Mice were handled according to the guidelines provided by the Canadian Council on Animal Care and the University of Saskatchewan Policy on Care and Use of Animals in Research. Protocols for the collection of mouse cells were approved by the University of Saskatchewan’s Animal Research Ethics Board (Surgical Procedures 19940211).

### Cell Culture

Cell lines and primary mouse cells are adherent, and were maintained in complete Dulbecco’s Modified Eagle Medium (DMEM) supplemented with 10% fetal bovine serum, 1% Penicillin/Streptomycin, and 1% non-essential amino acids as described in [26]. Huh7.5 cells were provided by Charles Rice (New York, USA) [55]. Wild-type mouse embryonic fibroblasts (MEFs) were obtained from Gregory Hannon (Cold Spring Harbor, USA), Robert Silverman [43], and Per Antonson (Huddinge, Sweden). NCoA6 knockout MEFs (also known as RAP250 knockout MEFs) were also provided by Per Antonson [48]. PKR knockout MEFs and PKR knockout mice were provided by John Bell (Ottawa, Canada) [42]. We isolated primary PKR knockout MEFs from the mice as in [56]. Briefly, a 13 to 15-day pregnant mouse was euthanized with isoflurane and embryos were extracted. Heads, appendages, and red matter were excised from the embryos, and the embryos were placed into a fresh dish. After chopping up the embryo tissue with scissors, it was incubated in trypsin for 1 hr at 37°C, before pelleting and plating in complete DMEM. PKR knockout hepatocytes were isolated as follows: a male 7 to 9 week-old PKR knockout mouse was euthanized with isoflurane, and the liver perfused from the vena cava with 25 mL 37°C Krebs Ringer with glucose and 0.1 mM EDTA. The liver was then digested with collagenase (12.6 mg) in 25 mL Krebs Ringer with glucose and 150 µM CaCl_2_ (Buffer 2). After digestion, the liver was removed from the body cavity and transferred to a petri dish, where it was punctured and resuspended in Buffer 2, and then filtered through a 70 µM filter and washed twice with Buffer 2. Cells were then tested for viability with trypan blue, and, if greater than 80% viable, seeded at a density of 2.0×10^5^ cells/well in collagen-coated 24-well dishes in complete DMEM.

### Viral Plasmids and RNA

Plasmids pSGR JFH-1 Fluc WT and pSGR JFH-1 Fluc GND encode sub-genomic JFH-1-derived HCV replicons with a firefly luciferase reporter; the GND contains an inactivating mutation in the viral polymerase [57], [58]. Plasmids pSGR S1+S2:p3 Fluc WT and pSGR S1+S2:p3 Fluc GND have C to G mutations at position 3 in both miR-122 seed binding sites in the HCV 5′ UTR and were described in [26]. Plasmids pJ6/JFH-1(p7-Rluc2a) “FL WT” and pJ6/JFH-1(p7-Rluc2a) GNN “FL GNN” bear full-length viral sequences derived from the J6 (structural proteins) and JFH-1 (non-structural proteins) isolates of HCV, and a Renilla luciferase reporter, with the GNN having inactivating mutations in the viral polymerase [59]. Firefly luciferase control mRNA was transcribed from Luciferase T7 Control DNA plasmid (Promega; Nepean, ON, Canada), while Renilla luciferase control mRNA was transcribed from the pRL-TK plasmid (Promega). Plasmid templates for viral RNA and mRNA were prepared and *in vitro* transcribed with the MEGAScript T7 High Yield Transcription Kit and mMessage mMachine T7 Transcription Kit (Life Technologies; Burlington, ON, Canada), respectively, as described in [26].

### microRNAs and Silencing RNAs

miR-122∶5′-UGG AGU GUG ACA AUG GUG UUU GU-3′ and miR-122*: 5′-AAA CGC CAU UAU CAC ACU AAA UA-3′, annealed. miControl: 5′- GAA GGU CAC UCA GCU AAU CAC and miControl*: 5′-GUG AUU AGC UGA CAG ACC UUC-3′, annealed [60]. siPKR: 5′-GCG AGA AAC UAG ACA AAG U-3′. siNCoA6∶5′-CCA CAG AGC UGG ACA GUA AUU-3′. siControl: 5′- GAA GGU CAC UCA GCU AAU CAC dTTC-3′ [61]. All small RNAs were synthesized by ThermoScientific Dharmacon (Lafayette, CO, USA).

### Electroporation

Cells were electroporated using 4 mm cuvettes at infinite resistance, and were prepared and plated as described in [26]. Briefly, cells were trypsinized, washed twice with cold Dulbecco’s PBS (D-PBS), then resuspended in D-PBS at a concentration of 1.5×10^7^ cells/mL, with 400 µL (6.0×10^6^) cells used per sample. Mouse cell lines were electroporated with 10 µg viral RNA, 1 µg control Fluc or Rluc mRNA as transfection control, and 60 pmol miRNA. Huh7.5 cells were electroporated with 5 µg viral RNA and 1 µg Rluc mRNA as transfection control. When used for knockdown, Huh7.5 cells were first electroporated with 60 pmol siRNA and incubated for three days; they were then electroporated again with 60 pmol siRNA, along with 5 µg viral RNA and 1 µg Rluc mRNA as transfection control. Electroporation conditions for MEFs were 400V, 250 µF; for PKR knockout hepatocytes were 220V, 950 µF; and for Huh7.5 cells were 270V, 950 µF. Cells were then resuspended in 4 mL DMEM, and 500 µL cells were plated in 6-well dishes for luciferase harvests, or 2 mL cells were plated into 10 cm dishes for RNA harvests.

### Transfection

Briefly, media was replaced with Pen/Strep-free C-DMEM on PKR knockout hepatocytes one day post-extraction. Lipofectamine 2000 reaction mixture was assembled according to recommended protocol, with 0.4 µg viral RNA, 5.0 pmol miRNA, and 0.1 µg mRNA assembled with 1.5 µL Lipofectamine 2000 (approximately 3∶1 ratio) in OptiMEM. Cells were transfected overnight, media was changed on Day 1 post-electroporation to C-DMEM, and luciferase was harvested as indicated.

### Luciferase Assays

Luciferase assays to monitor viral replication were carried out as described in [26]. Briefly, cells were harvested by scraping in 100 µL of passive lysis buffer. 10 µL of lysate was assayed for light production in 50 µL of the appropriate assay buffer (luciferase substrate) for the type of luciferase in the sample, according to the associated protocol, and using a GLOMAX luminometer (Promega).

### Infections

Naive Huh7.5 cells were plated at 1.0×10^5^ cells per well in a 6-well dish one day pre-infection. Supernatant from NCoA6 cells was collected at the indicated time, spun to pellet cell debris, and 2 mL was plated on naive Huh7.5 cells. Huh7.5 cell extracts were harvested as above at three days post-infection and assayed for luciferase expression to detect HCV infection.

### RNA Collection

Total RNA was collected from cells three days post-electroporation into 1 mL Trizol (Life Technologies) and isolated according to the manufacturer’s protocol.

### Northern Blot

Northern blotting for HCV RNA was carried out as described in [26].

### qRT-PCR

Total cellular RNA was reverse-transcribed using the iScript cDNA Synthesis Kit (BioRad; Missisauga, ON, Canada). qPCR reactions were carried out using the TaqMan kits Hs00169345_m1 (EIF2AK2, PKR), Hs01052843_m1 (NCoA6) and FAM-MGB 4352934-0803022 (GAPDH); samples were amplified in triplicate with 2X TaqMan Master Mix (Life Technologies). All data was analyzed with the CFX Manager Software (BioRad).

## References

[1] WHO (2012) Hepatitis C. World Health Organization Fact Sheet No.164.

[2] Strader DB, Wright T, Thomas DL, Seeff LB (2004) Diagnosis, management, and treatment of hepatitis C. Hepatology. 39: 1147–1171. 10.1002/hep.20119 15057920

[3] Bartenschlager R, Frese M, Pietschmann T (2004) Novel insights into hepatitis C virus replication and persistence. Adv Virus Res 63: 71–180. 15530561 10.1016/S0065-3527(04)63002-8

[4] Vogt A, Scull MA, Friling T, Horwitz JA, Donovan BM, et al.. (2013) Recapitulation of the hepatitis C virus life-cycle in engineered murine cell lines. Virology. 10.1016/j.virol.2013.05.036 PMC3755106 23777661

[5] Ploss A, Evans MJ, Gaysinskaya VA, Panis M, You H, et al (2009) Human occludin is a hepatitis C virus entry factor required for infection of mouse cells. Nature 457: 882–886. 19182773 10.1038/nature07684 PMC2762424

[6] Dorner M, Horwitz JA, Donovan BM, Labitt RN, Budell WC, et al (2013) Completion of the entire hepatitis C virus life cycle in genetically humanized mice. Nature 501: 237–241. 23903655 10.1038/nature12427 PMC3858853

[7] Dorner M, Rice CM, Ploss A (2013) Study of hepatitis C virus entry in genetically humanized mice. Methods 59: 249–257. 22687621 10.1016/j.ymeth.2012.05.010 PMC3652663

[8] Scarselli E, Ansuini H, Cerino R, Roccasecca RM, Acali S, et al (2002) The human scavenger receptor class B type I is a novel candidate receptor for the hepatitis C virus. Embo J 21: 5017–5025. 12356718 10.1093/emboj/cdf529 PMC129051

[9] Evans MJ, von Hahn T, Tscherne DM, Syder AJ, Panis M, et al (2007) Claudin-1 is a hepatitis C virus co-receptor required for a late step in entry. Nature 446: 801–805. 17325668 10.1038/nature05654

[10] Martin DN, Uprichard SL (2013) Identification of transferrin receptor 1 as a hepatitis C virus entry factor. Proceedings of the National Academy of Sciences 110: 10777–10782. 10.1073/pnas.1301764110 PMC3696786 23754414

[11] Sainz B Jr, Barretto N, Martin DN, Hiraga N, Imamura M, et al (2012) Identification of the Niemann-Pick C1-like 1 cholesterol absorption receptor as a new hepatitis C virus entry factor. Nat Med 18: 281–285. 22231557 10.1038/nm.2581 PMC3530957

[12] Lupberger J, Zeisel MB, Xiao F, Thumann C, Fofana I, et al (2011) EGFR and EphA2 are host factors for hepatitis C virus entry and possible targets for antiviral therapy. Nat Med 17: 589–595. 21516087 10.1038/nm.2341 PMC3938446

[13] Pileri P, Uematsu Y, Campagnoli S, Galli G, Falugi F, et al (1998) Binding of Hepatitis C Virus to CD81. Science 282: 938–941. 9794763 10.1126/science.282.5390.938

[14] Shi Q, Jiang J, Luo G (2013) Syndecan-1 serves as the major receptor for attachment of hepatitis C virus to the surfaces of hepatocytes. J Virol 87: 6866–6875. 23576506 10.1128/JVI.03475-12 PMC3676102

[15] Frentzen A, Kusuma A, Guerlevik E, Hueging K, Knocke S, et al.. (2013) Cell entry, efficient RNA replication, and production of infectious hepatitis C virus progeny in mouse liver-derived cells. Hepatology. 10.1002/hep.26626 23873628

[16] Chang KS, Jiang J, Cai Z, Luo G (2007) Human apolipoprotein e is required for infectivity and production of hepatitis C virus in cell culture. Journal Of Virology 81: 13783–13793. 17913825 10.1128/JVI.01091-07 PMC2168882

[17] Jopling CL, Norman KL, Sarnow P (2006) Positive and negative modulation of viral and cellular mRNAs by liver-specific microRNA miR-122. Cold Spring Harb Symp Quant Biol 71: 369–376. 17381319 10.1101/sqb.2006.71.022

[18] Jopling CL, Schutz S, Sarnow P (2008) Position-dependent function for a tandem microRNA miR-122-binding site located in the hepatitis C virus RNA genome. Cell Host Microbe 4: 77–85. 18621012 10.1016/j.chom.2008.05.013 PMC3519368

[19] Jopling CL, Yi M, Lancaster AM, Lemon SM, Sarnow P (2005) Modulation of hepatitis C virus RNA abundance by a liver-specific MicroRNA. Science 309: 1577–1581. 16141076 10.1126/science.1113329

[20] Esau C, Davis S, Murray SF, Yu XX, Pandey SK, et al (2006) miR-122 regulation of lipid metabolism revealed by in vivo antisense targeting. Cell Metab 3: 87–98. 16459310 10.1016/j.cmet.2006.01.005

[21] Norman KL, Sarnow P (2010) Modulation of hepatitis C virus RNA abundance and the isoprenoid biosynthesis pathway by microRNA miR-122 involves distinct mechanisms. J Virol 84: 666–670. 19846523 10.1128/JVI.01156-09 PMC2798415

[22] Bai S, Nasser MW, Wang B, Hsu S-H, Datta J, et al (2009) MicroRNA-122 Inhibits Tumorigenic Properties of Hepatocellular Carcinoma Cells and Sensitizes These Cells to Sorafenib. J Biol Chem 284: 32015–32027. 19726678 10.1074/jbc.M109.016774 PMC2797273

[23] Xu J, Zhu X, Wu L, Yang R, Yang Z, et al (2012) MicroRNA-122 suppresses cell proliferation and induces cell apoptosis in hepatocellular carcinoma by directly targeting Wnt/β-catenin pathway. Liver International 32: 752–760. 22276989 10.1111/j.1478-3231.2011.02750.x

[24] Narbus CM, Israelow B, Sourisseau M, Michta ML, Hopcraft SE, et al (2011) HepG2 Cells Expressing MicroRNA miR-122 Support the Entire Hepatitis C Virus Life Cycle. Journal of Virology 85: 12087–12092. 21917968 10.1128/JVI.05843-11 PMC3209320

[25] Kambara H, Fukuhara T, Shiokawa M, Ono C, Ohara Y, et al (2012) Establishment of a Novel Permissive Cell Line for the Propagation of Hepatitis C Virus by Expression of MicroRNA miR122. Journal of Virology 86: 1382–1393. 22114337 10.1128/JVI.06242-11 PMC3264374

[26] Thibault PA, Huys A, Dhillon P, Wilson JA (2013) MicroRNA-122-dependent and -independent replication of Hepatitis C Virus in Hep3B human hepatoma cells. Virology 436: 179–190. 23245472 10.1016/j.virol.2012.11.007

[27] Fukuhara T, Kambara H, Shiokawa M, Ono C, Katoh H, et al (2012) Expression of miR-122 facilitates an efficient replication in nonhepatic cells upon infection with HCV. J Virol 86: 7918–7933. 22593164 10.1128/JVI.00567-12 PMC3421686

[28] Chang J, Guo JT, Jiang D, Guo H, Taylor JM, et al (2008) Liver-specific microRNA miR-122 enhances the replication of hepatitis C virus in nonhepatic cells. J Virol 82: 8215–8223. 18550664 10.1128/JVI.02575-07 PMC2519557

[29] Lin LT, Noyce RS, Pham TN, Wilson JA, Sisson GR, et al (2010) Replication of subgenomic hepatitis C virus replicons in mouse fibroblasts is facilitated by deletion of interferon regulatory factor 3 and expression of liver-specific microRNA 122. J Virol 84: 9170–9180. 20592082 10.1128/JVI.00559-10 PMC2937658

[30] Nandakumar R, Finsterbusch K, Lipps C, Neumann B, Grashoff M, et al.. (2013) Hepatitis C Virus Replication in Mouse Cells is Restricted by IFN-Dependent and -Independent Mechanisms. Gastroenterology. 10.1053/j.gastro.2013.08.037 23973921

[31] MacArthur KL, Wu CH, Wu GY (2012) Animal models for the study of hepatitis C virus infection and replication. World J Gastroenterol 18: 2909–2913. 22736914 10.3748/wjg.v18.i23.2909 PMC3380318

[32] Mercer DF, Schiller DE, Elliott JF, Douglas DN, Hao C, et al (2001) Hepatitis C virus replication in mice with chimeric human livers. Nat Med 7: 927–933. 11479625 10.1038/90968

[33] Bility MT, Zhang L, Washburn ML, Curtis TA, Kovalev GI, et al (2012) Generation of a humanized mouse model with both human immune system and liver cells to model hepatitis C virus infection and liver immunopathogenesis. Nat Protoc 7: 1608–1617. 22899330 10.1038/nprot.2012.083 PMC3979325

[34] Robinet E, Baumert TF (2011) A first step towards a mouse model for hepatitis C virus infection containing a human immune system. J Hepatol 55: 718–720. 21616105 10.1016/j.jhep.2011.02.038

[35] Ploss A, Rice CM (2009) Towards a small animal model for hepatitis C. EMBO Rep. 10: 1220–1227. 10.1038/embor.2009.223 PMC2775186 19834510

[36] Wadman M (2013) Time called on chimp work. Nature 495: 289–290. 23518537 10.1038/495289a

[37] Xie ZC, Riezu-Boj JI, Lasarte JJ, Guillen J, Su JH, et al (1998) Transmission of hepatitis C virus infection to tree shrews. Virology 244: 513–520. 9601519 10.1006/viro.1998.9127

[38] Xu X, Chen H, Cao X, Ben K (2007) Efficient infection of tree shrew (Tupaia belangeri) with hepatitis C virus grown in cell culture or from patient plasma. J Gen Virol 88: 2504–2512. 17698660 10.1099/vir.0.82878-0

[39] Amako Y, Tsukiyama-Kohara K, Katsume A, Hirata Y, Sekiguchi S, et al (2010) Pathogenesis of hepatitis C virus infection in Tupaia belangeri. J Virol 84: 303–311. 19846521 10.1128/JVI.01448-09 PMC2798454

[40] Dabo S, Meurs EF (2012) dsRNA-dependent protein kinase PKR and its role in stress, signaling and HCV infection. Viruses 4: 2598–2635. 23202496 10.3390/v4112598 PMC3509664

[41] Chang KS, Cai Z, Zhang C, Sen GC, Williams BR, et al (2006) Replication of hepatitis C virus (HCV) RNA in mouse embryonic fibroblasts: protein kinase R (PKR)-dependent and PKR-independent mechanisms for controlling HCV RNA replication and mediating interferon activities. J Virol 80: 7364–7374. 16840317 10.1128/JVI.00586-06 PMC1563689

[42] Abraham N, Stojdl DF, Duncan PI, Méthot N, Ishii T, et al (1999) Characterization of Transgenic Mice with Targeted Disruption of the Catalytic Domain of the Double-stranded RNA-dependent Protein Kinase, PKR. J Biol Chem 274: 5953–5962. 10026221 10.1074/jbc.274.9.5953

[43] Yang YL, Reis LF, Pavlovic J, Aguzzi A, Schafer R, et al (1995) Deficient signaling in mice devoid of double-stranded RNA-dependent protein kinase. Embo J 14: 6095–6106. 8557029 10.1002/j.1460-2075.1995.tb00300.x PMC394734

[44] Kim S-W, Park K, Kwak E, Choi E, Lee S, et al (2003) Activating Signal Cointegrator 2 Required for Liver Lipid Metabolism Mediated by Liver X Receptors in Mice. Mol Cell Biol 23: 3583–3592. 12724417 10.1128/MCB.23.10.3583-3592.2003 PMC164762

[45] Li Q, Chu M-J, Xu J (2007) Tissue- and Nuclear Receptor-Specific Function of the C-Terminal LXXLL Motif of Coactivator NCoA6/AIB3 in Mice. Mol Cell Biol 27: 8073–8086. 17908797 10.1128/MCB.00451-07 PMC2169164

[46] Mahajan MA, Samuels HH (2005) Nuclear hormone receptor coregulator: role in hormone action, metabolism, growth, and development. Endocr Rev 26: 583–597. 15561801 10.1210/er.2004-0012

[47] Tai AW, Benita Y, Peng LF, Kim SS, Sakamoto N, et al (2009) A functional genomic screen identifies cellular cofactors of hepatitis C virus replication. Cell Host Microbe 5: 298–307. 19286138 10.1016/j.chom.2009.02.001 PMC2756022

[48] Antonson P, Schuster GU, Wang L, Rozell B, Holter E, et al (2003) Inactivation of the Nuclear Receptor Coactivator RAP250 in Mice Results in Placental Vascular Dysfunction. Mol Cell Biol 23: 1260–1268. 12556486 10.1128/MCB.23.4.1260-1268.2003 PMC141133

[49] Pacini L, Graziani R, Bartholomew L, De Francesco R, Paonessa G (2009) Naturally Occurring Hepatitis C Virus Subgenomic Deletion Mutants Replicate Efficiently in Huh-7 Cells and Are trans-Packaged In Vitro To Generate Infectious Defective Particles. J Virol 83: 9079–9093. 19587042 10.1128/JVI.00308-09 PMC2738267

[50] Lohmann V, Hoffmann S, Herian U, Penin F, Bartenschlager R (2003) Viral and cellular determinants of hepatitis C virus RNA replication in cell culture. J Virol 77: 3007–3019. 12584326 10.1128/JVI.77.5.3007-3019.2003 PMC149776

[51] Zhu Q, Guo J-T, Seeger C (2003) Replication of Hepatitis C Virus Subgenomes in Nonhepatic Epithelial and Mouse Hepatoma Cells. J Virol 77: 9204–9210. 12915536 10.1128/JVI.77.17.9204-9210.2003 PMC187424

[52] Uprichard SL, Chung J, Chisari FV, Wakita T (2006) Replication of a hepatitis C virus replicon clone in mouse cells. Virology Journal 3: 89. 17069661 10.1186/1743-422X-3-89 PMC1635043

[53] Aly HH, Oshiumi H, Shime H, Matsumoto M, Wakita T, et al (2011) Development of mouse hepatocyte lines permissive for hepatitis C virus (HCV). PLoS One 6: e21284. 21731692 10.1371/journal.pone.0021284 PMC3120852

[54] Frentzen A, Huging K, Bitzegeio J, Friesland M, Haid S, et al (2011) Completion of hepatitis C virus replication cycle in heterokaryons excludes dominant restrictions in human non-liver and mouse liver cell lines. PLoS Pathog 7: e1002029. 21552323 10.1371/journal.ppat.1002029 PMC3084199

[55] Blight KJ, McKeating JA, Rice CM (2002) Highly Permissive Cell Lines for Subgenomic and Genomic Hepatitis C Virus RNA Replication. J Virol 76: 13001–13014. 12438626 10.1128/JVI.76.24.13001-13014.2002 PMC136668

[56] Lewis JD, Meehan RR, Henzel WJ, Maurer-Fogy I, Jeppesen P, et al (1992) Purification, sequence, and cellular localization of a novel chromosomal protein that binds to Methylated DNA. Cell 69: 905–914. 1606614 10.1016/0092-8674(92)90610-o

[57] Kato T, Date T, Miyamoto M, Furusaka A, Tokushige K, et al (2003) Efficient replication of the genotype 2a hepatitis C virus subgenomic replicon. Gastroenterology 125: 1808–1817. 14724833 10.1053/j.gastro.2003.09.023

[58] Kato T, Date T, Miyamoto M, Sugiyama M, Tanaka Y, et al (2005) Detection of anti-hepatitis C virus effects of interferon and ribavirin by a sensitive replicon system. J Clin Microbiol 43: 5679–5684. 16272504 10.1128/JCM.43.11.5679-5684.2005 PMC1287837

[59] Jones CT, Murray CL, Eastman DK, Tassello J, Rice CM (2007) Hepatitis C virus p7 and NS2 proteins are essential for production of infectious virus. Journal Of Virology 81: 8374–8383. 17537845 10.1128/JVI.00690-07 PMC1951341

[60] Wilson JA, Zhang C, Huys A, Richardson CD (2011) Human Ago2 Is Required for Efficient MicroRNA 122 Regulation of Hepatitis C Virus RNA Accumulation and Translation. J Virol 85: 2342–2350. 21177824 10.1128/JVI.02046-10 PMC3067765

[61] Wilson JA, Jayasena S, Khvorova A, Sabatinos S, Rodrigue-Gervais IG, et al (2003) RNA interference blocks gene expression and RNA synthesis from hepatitis C replicons propagated in human liver cells. Proc Natl Acad Sci U S A 100: 2783–2788. 12594341 10.1073/pnas.252758799 PMC151418

